# The Application of Microelectromechanical Systems (MEMS) Accelerometers to the Assessment of Blast Threat to Armored Vehicle Crew

**DOI:** 10.3390/s22010316

**Published:** 2021-12-31

**Authors:** Sławomir Kciuk, Edyta Krzystała, Arkadiusz Mężyk, Paweł Szmidt

**Affiliations:** 1 Department of Theoretical and Applied Mechanics, Faculty of Mechanical Engineering, Silesian University of Technology, 44-100 Gliwice, Poland; Slawomir.Kciuk@polsl.pl (S.K.); Arkadiusz.Mezyk@polsl.pl (A.M.); 2 DUAL-PROJEKT Szmidt Paweł, 44-141 Gliwice, Poland; pszmidt@dual-projekt.com.pl

**Keywords:** blast load, mine-resistant vehicle, MEMS accelerometers, impulse measurement, measurement system

## Abstract

This paper describes the development and application of an autonomous register and measurement system (ARMS), and the application of microelectromechanical systems (MEMS) accelerometers to the assessment of blast threat to armored vehicle crews. Taking measurements with reference to an explosion is one of the principal issues in the protection of crews of special vehicles. The proposed ARMS reduces research costs and contributes to the development of an autonomous, wireless test stand, applicable in various research areas and industry. The ARMS performs data acquisition with simultaneous measurement in multiple channels. The maximum sampling rate is 100 kHz and the sensor range is ±500 g. This solution is an alternative to cable systems, which have a high energy demand. The functionality of the developed autonomous measuring system is demonstrated experimentally. The paper concludes with a field study of the proposed system and the application of MEMS accelerometers via a mine blast test of a military vehicle at level 4 of STANAG 4569.

## 1. Introduction

The research and development of modern military vehicles is primarily concerned with reducing vehicle weight while ensuring a high level of crew protection and high operational parameters. This approach is dictated by the nature of modern combat operations which involve the participation of mobile troops in stabilization missions or military operations in various parts of the world. Such operations require vehicles with relatively low mass and high firepower, while displaying maximum survivability on the battlefield [1,2,3]. 

The protection of crews of military vehicles became a pre-eminent issue in NATO countries with the emergence of the first analyses and consequences of military operations in Iraq and Afghanistan. Based on the statistics of contemporary military operations or stabilization missions, it is estimated that in approximately 60% of cases, mines and improvised explosive devices (IEDs) are the principal cause of death or serious injury to soldiers [4,5,6,7,8,9,10].

The tactics used by terrorist groups to conduct irregular and guerrilla operations, the primary target of which being moving vehicles and their crews, has encouraged the introduction of vehicles that provide protection to soldiers from shock waves generated by the detonation of mines and IEDs.

The mine-resistant ambush protected (MRAP) program was created in the United States of America in early 2007 in response to the military’s demand for explosive-proof vehicles. The use of mine-resistant vehicles resulted in a significant decrease in casualties among soldiers participating in combat operations.

An important consideration when designing anti-mine protection measures is the use of synergistic research methods to obtain optimal geometric features and ensure the desired operating parameters are achieved. The basis for the development of modern mine protection measures should be the identification of the shock wave impact on not only the vehicle structure, but, primarily, the crew. Tests identifying the overload acting on the crews of military vehicles are carried out in accordance with the mine protection standards within AEP 55 Volume 2, STANAG 4569 and HFM-090/TG-25 [1,2,3].

Standard criteria for assessing the risk to life of the crew apply to those parts of the body classified as most vulnerable to injuries: lower limbs, thoracic-lumbar spine, cervical spine and head, and chest and internal organs [4]. Analysis of data from the wars in Afganistan and Iraq shows that soldier mortality was most frequently associated with brain injury, and the most frequently injured part of the body were the lower limbs, resulting in amputation or fracture [4,5,8,9,10]. 

The ability to take measurements during an explosion is a highly important issue for the protection of crew in special-purpose vehicles. Such measurements require the application of conducting systems with a large energy demand. The conditions in which the research takes place can lead to wires within the equipment becoming disconnected, resulting in the disruption of signal canvassing and loss of data. In addition, research objects and measuring systems can be destroyed [1,2,3,4,5,6,11,12,13,14,15].

Here, we describe the development of an autonomic system (ARMS) to perform measurements and acquire data. The resultant data is used to identify parameters describing crew overload at the instant of charge explosion beneath a wheeled special-purpose vehicle. Field tests, which use acceleration sensors mounted at characteristic points on a dummy, are undertaken to identify the crew overload in the most exposed parts of the body. The vertical accelerations affecting the lower limbs and the pelvis, in addition to the pressure affecting the chest and the hearing aid are measured. A dedicated, autonomous measurement system is used to record acceleration signals at selected points on the bodies of the crew to determine their biomechanical parameters [4,12].

A test stand was developed, which enables the simultaneous measurement of both the acceleration of the seats and bodies of the crew, and the pressure acting on the bodies of the soldiers due to the explosion beneath the vehicle. The test stand includes a blast mitigation seat, an anthropomorphic mannequin weighing 75 kg, a set of microelectromechanical systems (MEMS) accelerometer sensors, and unique equipment for the acquisition of measurement data.

Due to its functionality, low purchase cost and high technical parameters, the developed system is appropriate for the validation or verification of models in the design and operation process. The measurement data obtained by the ARMS could be used to verify and optimize special-purpose structures, and thus directly impact the improvement of human safety.

The continuous development and miniaturization of MEMS sensors introduces new possibilities for their use. The literature documents the use of a triaxial accelerometer for medical diagnostics in a device tracking patient movement [16,17,18]. Multiple examples exist of the application of high-precision MEMS accelerometers in the army and in the civilian field [16,17,19,20]. Research has been undertaken on high-performance integrated circuits for closed-loop applications [16,17]. MEMS piezoresistive accelerometers are often used due to their low cost, easy implementation and simple detection electronics [21,22]. Acar and Shkel [21] present an analysis of commerical MEMS accelerometers with variable capacity. Capacitive MEMS sensors with the same low-level input acceleration range and varied mechanical sensing element designs, materials, fabrication technologies, and price ranges are analyzed. Four sensors were selected, and characteristics of interest included sensitivity, resolution, linearity, frequency response, transverse sensitivity, reaction to temperature, noise level, and long-term stability. Davis [22] and Ragam and Nimaje [23] present information on the potential use of MEMS sensors in the military. Test results show that many such sensors are sufficiently robust to survive the demanding application conditions [22]. Moreover, these devices have been implemented in many commercial applications, such as automotive airbags, navigation, and instrumentation [21]. Conventional systems are limited due to being wire-based, expensive, and unable to transfer real-time information seamlessly [24,25,26].

Military operations in Iraq and Afghanistan have resulted in the increased exposure of military personnel to explosive threats. Injury prediction for these events is challenging due to the limited availability of blast-specific test studies and the use of established automotive-based injury criteria that do not directly translate to combat-related exposures [10].

Traumatic brain injury is a severe condition, for which the main cause is excessive mechanical loading during vehicle accidents, sports injuries, violence, or injuries related to everyday activities (e.g., impact with furniture or falls down stairs) [27,28,29,30,31]. Asymmetry within military action zones characterizes actions which differ from those of a stronger enemy, the aim of which being to increase dominance, exploit the weaknesses of the stronger enemy, seize initiative, and gain increased freedom of action [32,33,34,35]. The primary mechanism of a lower limb injury was examined by Ramasamy, who discovered that such injuries are the result of limb motion or contact between the limb and elements within the environment, and are commonly referred to as third-rate damage from the blast [4,12]. The fracture characteristics include three-point bending, spiral fractures and long bone fractures directly related to the axial load. In the case of smaller impulse loads, the injuries more frequently take the form of micro-injuries and soft tissue injuries [36]. 

The estimation of a crew’s endangerment due to an explosion beneath a military vehicle is a complex and difficult task that depends on many mutually dependent factors. Thus, the estimation of life and health endangerment should be based on a complex analysis of such an event [4,12].

## 2. Materials and Methods

System Development

**Figure 1:**
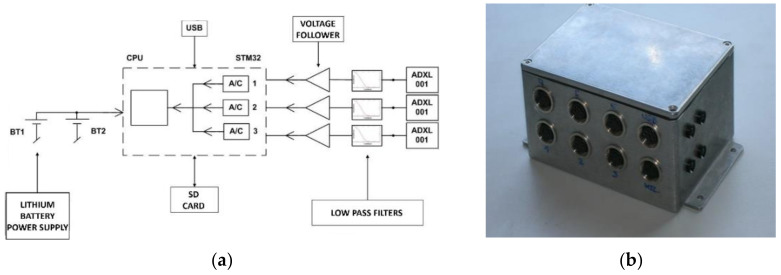
(**a**) ARMS architecture and (**b**) physical form.

**Figure 2:**
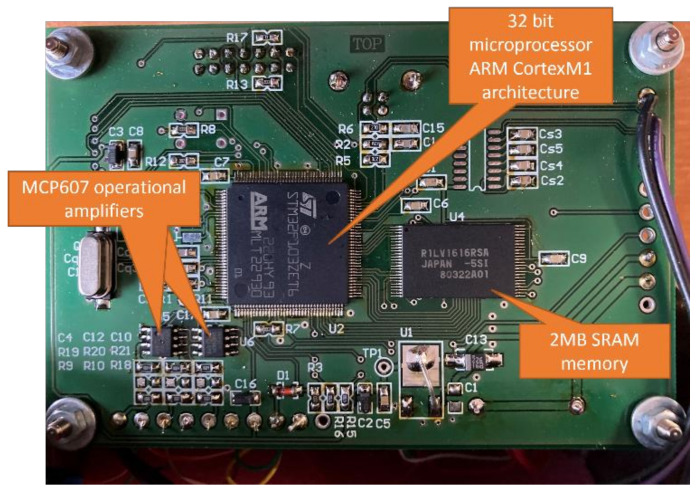
Physical form of the ARMS.

The autonomous measurement system (ARMS) (Figure 1) was developed at the Department of Theoretical and Applied Mechanics within the Faculty of Mechanical Engineering, Silesian University of Technology (Table 1). The system consists of (Figure 2) data collection and general postprocessing, and performs data acquisition with simultaneous measurement in two, four, six or eight channels. The maximum sampling rate is 100 kHz. The resolution is 2.5 m/s^2^ and the sensor range is ±500 g. Using a pretrigger, the ARMS can record signals in advance of the event under investigation.

Measurement data is saved in real time on SRAM, after ending the measurement process, then processed (filtered), exported to file and saved on memory SD card (Figure 2). After this it is analyzed via the user interface. This feature is crucial when taking measurements during an explosion or similarly difficult environmental conditions, when the measuring equipment may be damaged or the measuring path may be destroyed at any time. The measuring system used is an innovative method of data acquisition, which is adapted to the most extreme test conditions.

The ARMS functions as follows. The signal from the measuring sensor is fed through the low-pass filter to a voltage follower composed of operational amplifiers. The signal from the voltage repeater is fed directly to the measuring input of the analog-to-digital converter. Each measuring input has a separate, independent transducer. This transducer is an integral part of the microprocessor. This solution enables the measurement of all input signals simultaneously. Signals are measured at 100 kHz in each channel, requiring 10 µs sample spacing. The processed signal value from each channel is stored in an independent buffer with a capacity of 300,000 samples. 

When the measurement is triggered, a marker is written to the buffer to indicate the beginning of the actual data. Concurrently, a sample counter is initiated. After 290,000 samples have been counted, processing stops. The remaining 10,000 samples are taken from the signals measured for 100 ms before the trigger. The measurement in all channels is triggered simultaneously by shorting to ground. The triggering connector, during the polygon tests described below, was connected to the pyrotechnic bus, so, along with the ignition initiation, the measurements were triggered. Following processing, data from measurement buffers are saved in text form onto a memory SD card. Once the data are saved, the ARMS can be connected to a computer and the file read from memory.

The developed system has a simple structure, reduces research costs, and, most importantly, contributes to the development of an autonomous, wireless test stand that can be applicable in various research areas and industry. This solution presents an alternative to the use of high budget non-autonomous cable systems or similar measurement systems, which have a high energy demand. The developed solution can be used in difficult environmental conditions, and due to the developed recording system, can acquire measurement data in several channels simultaneously. Measurement data is obtained via an autonomous system (ARMS) for the acquisition of rapidly changing voltage signals and is used to verify and optimize special-purpose structures, thus directly improving human safety.

Due to the use of a battery, the ARMS does not require an external power supply, such as a generator, that may adversely affect the natural environment. The ARMS has small physical dimensions and low weight.

## 3. System Application

This section describes the application of the autonomous measurement system (ARMS) and MEMS accelerometers to assess the threat to the crews of special vehicles during an explosion. Based on cooperation of our interdisciplinary research team, we were able to carry out military field research. The primary goal of the research was to verify the explosive resistance of the special wheeled vehicle and the blast mitigation seat, and to identify the load on the crew.

The tests were carried out on a prototype of a wheeled MRAP All-Terrain Vehicle M-ATV G-10 class, weighing 12.5 tons, manufactured by Dom Samochodowy GERMAZ Ltd. The vehicle, depending on the attached equipment, is designed to transport ten crew members or perform specialized tasks related to combat and security operations on the battlefield. The M-ATV G-10 has a self-supporting body made of armor plate, built on the chassis of a Mercedes Unimog U 500. The side walls of the vehicle are inclined in the shape of a diamond, giving the vehicle increased ballistic resistance to bullet penetration. In addition, the vehicle has low-resistance due to the V-shaped floor of the crew compartment integrated with the self-supporting body. This floor shape also helps to dissipate the energy of the blast shock wave generated by a mine or IED explosion.

**Figure 3:**
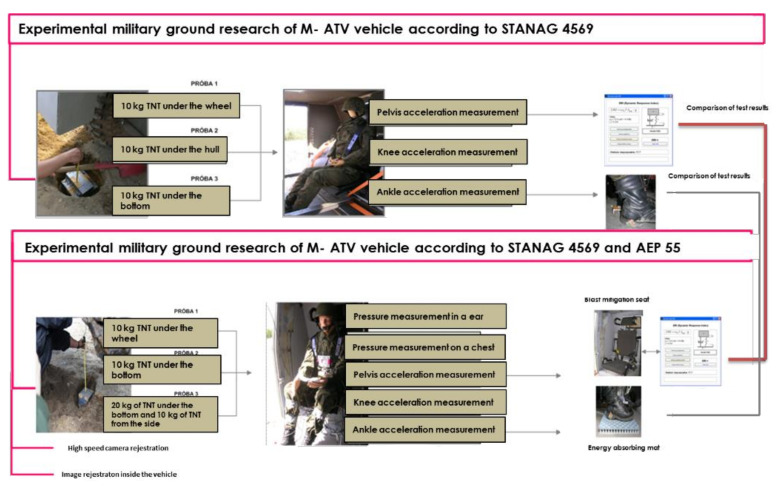
Overview of the experimental research.

To verify the vehicle’s mine resistance, two sets of field tests were performed. The purpose of the first, conducted at a training ground in Żagań, was to verify the mine resistance of the prototype vehicle and identify the impact of the shock wave on the bottom of the vehicle and the crew. The purpose of the second set of tests, conducted at an artillery training ground in Świętoszów, was to additionally verify the reliability of the protective measures used within the vehicle, such as a specialist blast mitigation seat and an underfoot anti-explosion mat. Moreover, using the developed, prototype test stand, the range of pressure acting on the crew within the vehicle was measured. Figure 3 presents an overview of the experimental research.

During each of the two sets of tests, three controlled detonations of the TNT charge beneath the vehicle were carried out. During the first set of tests (B1), the three controlled detonations were 10 kg of TNT under the wheel (B1_P1), 10 kg of TNT under the center of the hull (B1_P2), and 10 kg of TNT under the reducer (B1_P3). An anthropomorphic dummy was used to analyze the impact of the location of the explosive charge on the threat to the crew, identify overloads on the most vulnerable parts of the soldiers’ bodies, and estimate the time of the overloads threatening the crew of the vehicle during the explosion.

**Figure 4:**
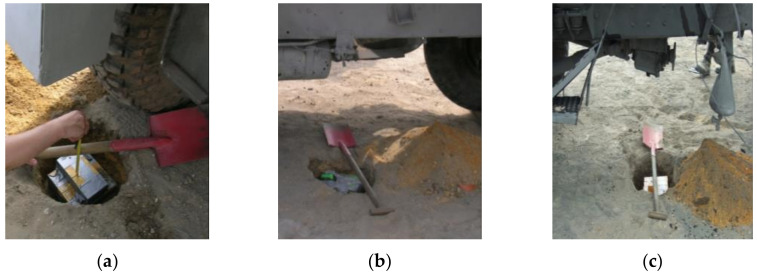
The load of TNT and its distribution during the first tests: (**a**)10 kg of TNT under the wheel, (**b**) 10 kg of TN under the center of the hull, (**c**) and 10 kg of TNT under the reducer.

During the second set of tests (B2), the following tests were carried out: 10 kg of TNT under the wheel(B2_P1), 10 kg of TNT under the center of the hull (B2_P2) (in accordance with the requirements of STANAG 4569). Figure 4 shows the load and its distribution during the tests. 

**Figure 5:**
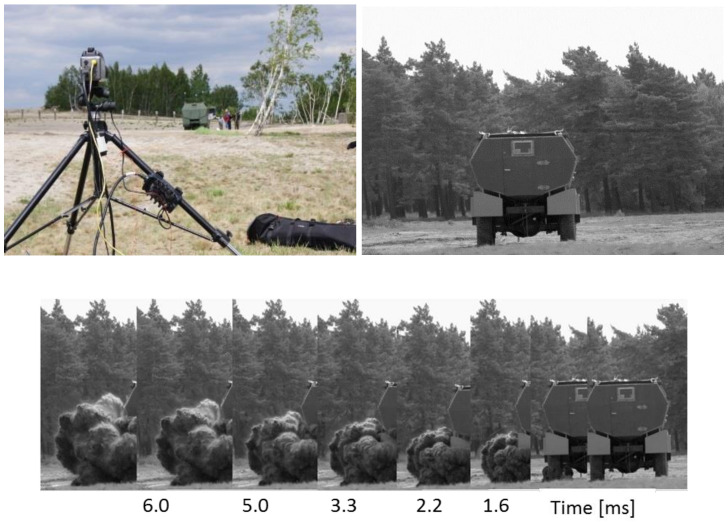
Image recording with high-speed cameras.

In addition, the blast mitigation seat was verified and the impact of the underfoot explosion mitigation mat on minimizing the overload on the lower limbs was verified. The pressure acting on the crew was measured using the developed test stand and a prototype autonomous measuring apparatus (ARMS). A novel contribution of this work was the introduction of an additional criterion for estimating the level of threat to the crew: the measurement of the pressure acting on the ear. During the experiments, images showing the propagation of the post-blast wave (Figure 5) were recorded using high-speed Phantom v9.1 cameras. In addition, images inside the vehicle during the explosion were recorded, enabling the analysis of the crew kinematics during the explosion.

**Figure 6:**
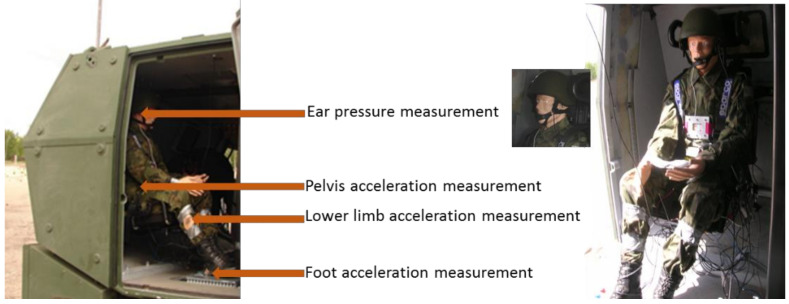
Research test stand presenting the points of attachment of MEMS acceleration and pressure sensors on the dummy.

The crew load was measured using MEMS accelerometers. During the tests the vertical accelerations affecting the lower limbs and pelvis, in addition to the pressure affecting the chest and the ear, were measured. Figure 6 shows the research test stand with attached MEMS accelerometers and autonomous measurement system (ARMS).

The tests were conducted in accordance with the mine protection standards AEP 55 Volume 2, STANAG 4569 and HFM-090/TG-25. A dedicated, autonomous measurement system was used to record acceleration signals at selected points on the dummy and to determine the biomechanical parameters of the crew.

**Figure 7:**
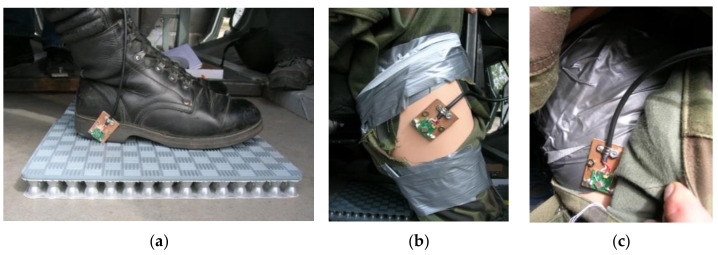
Measurement points on the dummy with attached MEMS accelerometers: (**a**) feet, (**b**) lower leg, (**c**) pelvis.

To perform the tests, a test stand was developed. This allows simultaneous measurements of both the acceleration of the dummy and the seat, and the pressure affecting the body of the dummy. The test stand (Figure 6) includes a blast mitigation seat, an anthropomorphic mannequin weighing 75 kg, a set of sensors (Figure 7), and unique equipment for the acquisition of measurement data. During the tests, MEMS acceleration sensors (Figure 7) were attached to the lower limbs (i.e., the foot and the lower leg) and the pelvis. Using the developed recording system, measurement data was acquired in multiple channels simultaneously.

This article provides selected examples of recorded signals to illustrate the capabilities of ARMS.

**Figure 8:**
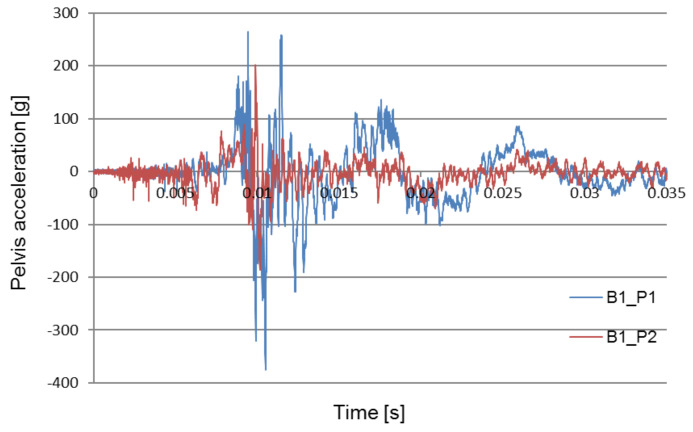
Comparison of two pelvis acceleration signals without the use of the anti-explosive seat.

**Figure 9:**
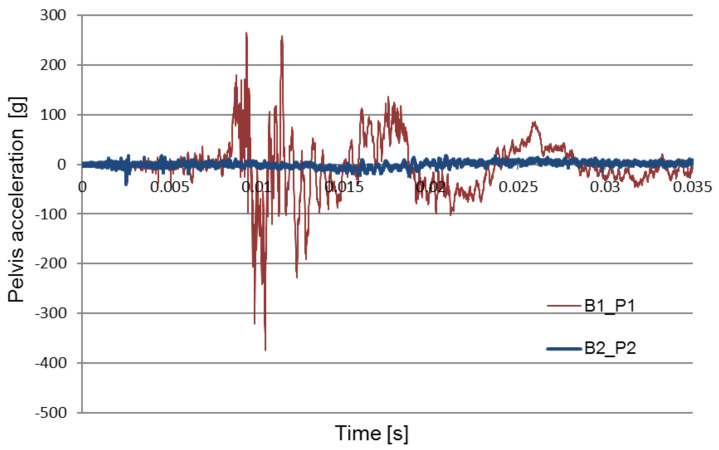
Comparison of two pelvis acceleration signals without (B1_P1) and with (B2_P1) use of the anti-explosive seat.

**Figure 10:**
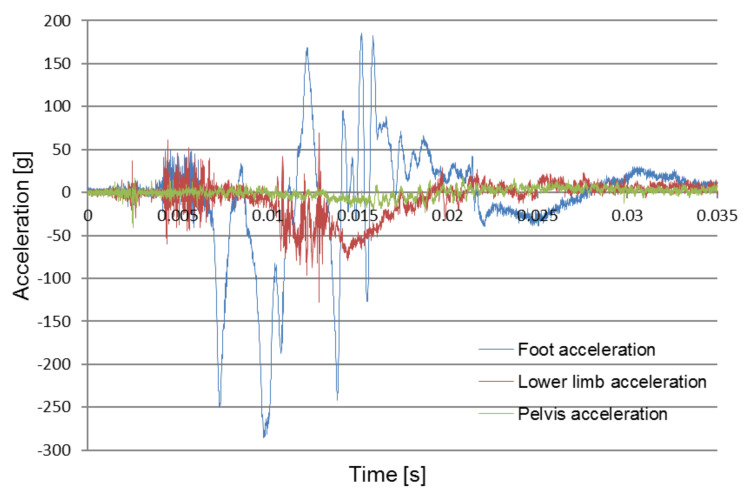
Comparison of recorded signals for pelvic and lower limb acceleration during the first test (B2_P1).

Figure 8, Figure 9 and Figure 10 compare the acceleration of the pelvis and the lower limbs with and without the use of protective measures. The obtained data forms the basis for the verification and optimization of the applied design solutions.

Figure 10 compares the recorded foot, pelvis and lower limb acceleration signals during the 10 kg of TNT explosion under the wheel (B2_P1) with the used blast mitigation seat as well as energy absorption mat under the foot. From the graphs, it can be concluded that for the lower limb (foot and tibia) the times of the first impulse coincide. However, the pelvic acceleration is reduced in comparison and does not show significant change.

**Figure 11:**
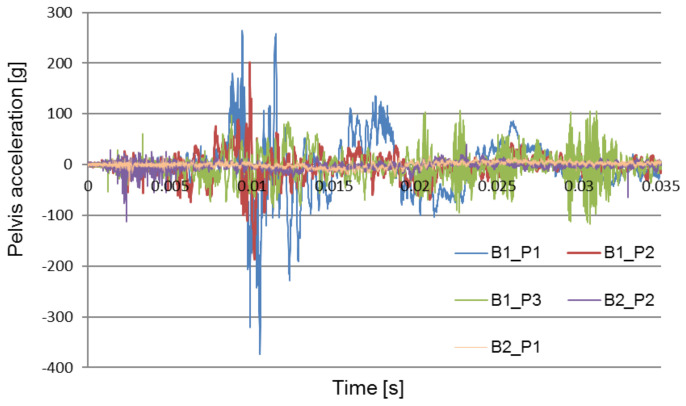
Comparison of pelvic acceleration signals recorded during the tests at the training grounds in Żagań (B1) and Świętoszów (B2).

Figure 11 shows a comparison of the pelvic acceleration waveforms recorded during two of the field experiments: the first without the use of the explosion-proof chair and the second with the use of the chair to absorb the explosion energy. The waveforms of the test signals performed in various conditions were selected for the analysis: a seat mounted above the wheel, in the middle of the fuselage.

The purpose of the analysis was to determine the time at which the pelvic acceleration impulse occurred. The graphs show that in the tests carried out without the blast mitigation seat the impulse occurs at approximately 0.01 s, while during the tests with the use of the blast mitigation seat these accelerations were reduced to safe values, and the first impulse appears at approximately 0.005 s.

**Figure 12:**
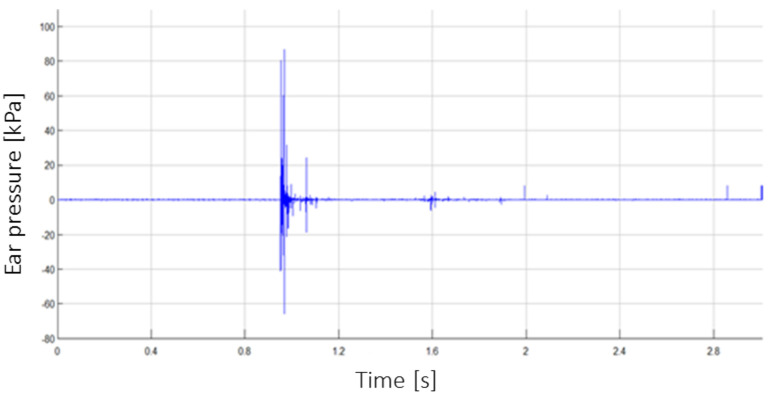
The ear pressure registered during detonation of 10 kg TNT under the wheel (B2_P1).

Figure 12 shows an example of the result of the pressure recorded in the ear during the detonation of 10 kg of TNT under the wheel of the vehicle (B2_P1). The attachment point of the KS-375M-100G pressure sensor is shown in Figure 6.

## 4. Discussion 

This study concerns the increase in effectiveness of anti-mine protection to suit the requirements of modern battlefields. Novel research is demonstrated on the identification of shock waves acting on the crew of a military vehicle. This research method and approach to the topic of crew protection is effective because it combines the two most important parameters, acceleration and pressure. The methodology, unique measuring system (ARMS), and results presented here may constitute a base verification of the factors which determine the level of a crew’s endangerment. Two patent applications were submitted to the Patent Office of the Republic of Poland concerning the developed equipment. The use of proprietary solutions for measuring systems (ARMS) contributed to the low cost and simplicity of the research procedures. The problem of protecting soldiers against a rapid pressure increase within a vehicle remains an unsolved problem.

Figure 8 compares pelvis acceleration signals registered without an anti-explosive seat during each of the two explosions: under the wheel, and under the center of the chassis. Each of the two attempts was made using the same amount of explosives. The contrast between the acceleration signals suggests that the highest maximum acceleration impulse was generated by the under-wheel explosion. Figure 9 compares types of pelvis acceleration recorded using the anti-explosive seat during the under-wheel explosion. The comparison between pelvis acceleration signals without (B1_P1) and with (B2_P1) for the use of the anti-explosive seat for the same test parameters and loading mass is presented in Figure 11. The presented results show that the greatest crew overload was registered during the under-heel explosion without the use of the anti-explosive seat. 

With the use of the special-purpose seat, the pelvis acceleration was reduced from approximately 280 g to 40 g and 100 g in the second attempt. 

The results and subsequent analysis demonstrate the efficacy of the anti-explosive seat and floor mat in the context of minimizing the crew’s overload. The results show that, with use of the anti-explosive seat, pelvis acceleration can be reduced to an acceptable value (Figure 9).

## 5. Conclusions

This paper presented an autonomous system (ARMS) for acquiring measurement data concerning accelerations acting on a test object as a result of impulse, shock, seismic waves, and other impacts. Measurement data is saved in real time to SRAM memory, and subsequently processed (filtered) and exported to a file and saved on memory SD card for analysis via a user interface. The use of a proprietary measurement system significantly reduces experimental research costs. Technical parameters include a high sampling frequency of 100 kHz in each channel and a measuring range of up to 500 g. Functionality, autonomy, and mobility present the measuring system as a strong alternative to wired systems with high energy demands. The developed system is a dual-use device; it can be applied in both civil and military scenarios. The measurement data obtained by the autonomous system are used to verify and optimize special-purpose structures, and thus have a direct impact on the improvement of human safety. 

This system (ARMS) has the potential to be used in aerospace, defense, energy, automotive, electronics, civil engineering, mining and commercial industrial applications. The presented solution allows the optimization of structures or investments (e.g., transportation) by extending the service life of such devices or improving their safety of use.

## 6. Patents

The ARMS described here has received patents from the Patent Office of the Republic of Poland, with patent numbers PL219525 (“Autonomous measuring system for fast-changing acquisition voltage signals”) and PL 221705 (“A method of autonomous acquisition of fast-changing voltage signals”). 

## Figures and Tables

**Table 1:** Device specifications.

Feature	Description
Acceleration sensor ADXL001	Range ± 500 g; Sensitivity: 3.3 mV/g; Noise: 70 mg rms; Noise Density: 2.76 mg/√Hz
Microprocessor STM32F103ZET6 ARM CortexM1 architecture	up to 1 MB program memory size 32-bit data bus width 12-bit ADC resolution up to 72 MHz maximum clock frequency up to 112 I/Os up to 96 KB data RAM size 2 V to 3.6 V operating supply voltage
MCP607 operational amplifiers	Bandwidth: 155 kHz Open-loop gain: 115 dB Mounting: THT Number of channels: 2 Case: DIP8 Slew rate: 80 mV/μs Quiescent current: 25 µA Input offset voltage: 250 µV Integrated circuit features: rail-to-rail output Operating voltage: 2.5–5.5 V
R1LV1616RSA SRAM memory	2 MB
Power supply	12 V
Dimension	110 × 100 × 160 (W × H × L) mm ok
Weight	0.45 kg

## Data Availability

Not applicable.
